# Folic acid ameliorates depression-like behaviour in a rat model of chronic unpredictable mild stress

**DOI:** 10.1186/s12868-020-0551-3

**Published:** 2020-01-15

**Authors:** Yue Zhou, Yu Cong, Huan Liu

**Affiliations:** 1 Department of Nutrition and Food Science, School of Public Health, Tianjin Medical University, No 22 Qixiangtai Road, Heping District, Tianjin, 300070 China; 2 Tianjin Key Laboratory of Environment, Nutrition and Public Health, Tianjin, China; 3 Tianjin Medical University Chu Hsien-l Memorial Hospital, Tianjin, China

**Keywords:** Folic acid, Depression-like behaviour, Chronic unpredictable mild stress (CUMS)

## Abstract

**Background:**

Depression is characterized by significant and low mood. Classical antidepressants are still not adequate in treating depression because of undesirable side effects. Folic acid, a member of the vitamin B complex, in considered to be strongly associated with the function and development of the central nervous system. Thus, in this study, we established a model of depression through chronic unpredictable mild stress (CUMS) in rats and assessed the antidepressant effects and mechanisms of folic acid.

**Methods:**

Sprague–Dawley rats were randomly divided into four groups: control, chronic unpredictable mild stress (CUMS), CUMS treated with folic acid, and CUMS treated with citalopram. Rats were assessed in terms of weight change, open-field test and sucrose preference. Homocysteine, monoamine neurotransmitters, interleukin-6, brain-derived neurotrophic factor (BDNF), β-endorphin levels in the serum and brains of rats were analysed.

**Results:**

Folic acid exhibited antidepressant-like effects in open-field and sucrose preference tests. Folic acid treatment effectively increased the levels of monoamine neurotransmitters, BDNF and β-endorphin, interleukin-6 and homocysteine levels were also significantly suppressed by folic acid administration.

**Conclusions:**

These findings serve as preclinical evidence that folic acid plays an antidepressant-like role in several pathways involving monoamine neurotransmitters. Thus, folic acid may be used as a potential antidepressant.

## Background

Depression is a widespread chronic medical illness that can affect thoughts, mood, and physical health [1]. The lifetime risk of depression is about 15–18%, meaning almost 1 in 5 people will experience a depressive episode at some point in their lifetime [2]. Among those diagnosed, many fail to achieve remission after following recommended antidepressant medication regimes and psychosocial therapies [3]. Studies have demonstrated that the core mechanism of depression is not clear from currently available treatments, and that we should seek new solutions for the treatment of depression. A meta-analysis for the association of folate and depression showed that folate in nutritional adjuvants received widespread attention in the treatment of depression [4].

There is accumulating evidence that low folate status is associated with depression. Depressed patients with low levels of folate have a lower likelihood of responding to antidepressant treatment, a greater likelihood of relapse and worse cognitive performance [5]. Another meta-analysis supports the relationship between low folate status and depression [6].

Whether long-term folic acid (FA) supplementation can reduce the risk of depression is controversial. A randomized controlled trial showed that long-term, high-dose, daily supplementation with FA, vitamin B6, vitamin B12 could not reduce the overall depression risk in middle aged and older women [7]. In several animal experiments, FA supplement alone [8] or combined with other nutrients (-zinc [9] or omega-3 fatty acids [10]) exerted significant antidepressant effects on rats.

The main mechanism of depression is described by the monoamine neurotransmitter hypothesis. This hypothesis may be supported by clinical observations dating back to the 1950s that reserpine, which depletes central stores of monoamines, can induce depression in a subset of patients [11, 12]. Folate is intimately linked to the synthesis of neurotransmitters in the central nervous system, such as serotonin (5-HT), norepinephrine (NE) and dopamine (DA) [13]. Furthermore, brain-derived neurotrophic factor (BDNF) levels is a useful marker for clinical response or improvement of depressive symptoms [14]. Lower serum β-endorphin (β-EP) levels were observed in a premenopausal depression model [15]. Interleukin-6 (IL-6) levels and depression are positively associated in clinical studies [16]. These indicators are all related to the synthesis and utilization of monoamine neurotransmitters.

Therefore, we established a model of depression through chronic unpredictable mild stress (CUMS) in rats and observed an antidepressant effect of FA. The levels of monoamine neurotransmitters, BDNF, β-EP and IL-6 in both serum and brain tissue were detected to explore the possible mechanism of action.

## Methods

### Animals

Male Sprague-Dawley (SD) rats weighing 220–250 g were purchased from the Beijing Vital River Laboratory Animal Technology (Beijing, China). All rats were housed with free access to food and water under a 12/12 h light/dark cycle at 21 °C ± 2° and 55% ± 10%-humidity. After being housed, rats were acclimated to their surroundings for 7 days to habituate to the experimenter. The animal experiment was performed according to the National Institutes of Health Guide for the Care and Use of Laboratory Animals and was approved by the Animal Use and Protection Committee of Tianjin Medical University. Furthermore, this manuscript reporting adheres to the ARRIVE guidelines for the reporting of animal experiments. We made every effort to minimize the number of animals used. The experimenters were blinded to the pharmacological treatment while processing data. Rats were euthanized by decapitation when the experimental procedure was finished.

### Chronic unpredictable mild stress (CUMS) procedure and experimental design

**Fig. 1:**
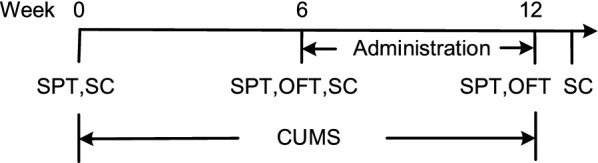
Animal treatment and experimental procedure. Animals were divided into four groups (Control group; CUMS group; CUMS + FA group; CUMS + CIT group). *CUMS* chronic unpredictable mild stress, *SPT* sucrose preference test, *OFT* open field test, *SC* sample collecting

All stressors for CUMS are shown in Table 1. To make the procedure unpredictable, 2 stressors were randomly applied each day. The entire experimental procedure is shown in Fig. 1.

**Table 1:** The stressors of CUMS procedure

Stressor	Duration
Food deprivation	24 h
Water deprivation	24 h
Illumination overnight	12 h
Physical restraint	2 h
Wet cage	8 h
Noise	10 min
Forced swimming at 4 °C	8 min
Tail pinch	2 min

After the adaptive feeding of rats, they were randomly divided into 4 groups of 10 rats each according to body weight: control, CUMS, CUMS + FA and CUMS + Citalopram (CUMS + CIT) groups.

CUMS model was considered successful when there were significant differences between the control and CUMS group in sucrose preference after 6 weeks of stimulate. Then, they received treatment respectively for the next 6 weeks within continued CUMS stimuli. The control group had no CUMS stimuli throughout the entire procedure.

FA (Sigma-Aldrich; St. Louis, MO) was administered intragastrically daily to CUMS + FA group using metal gavage needles at a volume of 0.8 mg/kg body weight. Citalopram (Sigma-Aldrich) was administered at 10 mg/kg in CUMS + CIT group. All other rats were an equal volume (10 mL/kg) of double-distilled water.

### Sucrose preference test

Before the experiment, rats were trained to drink sugary water in a noisy and quiet room for 1 day. Two 500 mL water bottles containing 2% sucrose water were placed in each cage at the same time. On the second day of the experiment, 2% sucrose water and 500 mL pure water were given to each rat in 500 mL/bottles. On the third day, the rats underwent fasting and water deprivation were observed. On the fourth day, the sucrose water consumption experiment was carried out in animals. At the same time, 2% sucrose water and 100 mL pure water in 100 mL/bottles were given to each rat. After 2 h, both sucrose water consumption and pure water consumption were measured, and the sucrose water preference of the animals was calculated Sucrose preference [%)] = sucrose consumption/(sucrose consumption + water consumption).

### Open field test (OFT)

The open field test was performed to measure spatial exploration behaviour. Test was performed using the OFT-100 open field test system (Chengdu Taimeng Technology Co. Ltd; St. Chengdu, China). Briefly, an apparatus consisting of a black square cage (62.5 cm × 74 cm × 451 cm) was divided into 3 × 3 equal small squares on the floor. A single rat was placed in the centre of the cage, and then locomotion was recorded for 5 min by measuring the total standing time and walking distance by video. The cage was completely cleaned with 90% alcohol after each test.

### FA and HCY measurement

The blood of femoral artery was taken after the intervention and was collected into heparinized tubes and centrifuged at 3000 rpm for 15 min at 4 °C.

FA assay: FA levels in serum were measured with an automated chemiluminescence system (Siemens Immulite 2000 Xpi; St. Berlin and Munich, Germany) using a competitive protein binding assay, according to the manufacturer’s instructions. This system detected all types of folate with a detection sensitivity limit of 0.8 ng/mL.

Hcy assay: Serum Hcy concentration was quantified by an enzymatic cycling method. Serum samples were mixed with Hcy Reagent (Meikang Medical System; St. Sichuan, China) in a reaction cell, then the absorbance was measured at 340 nm by an Auto-Chemistry Analyzer (DIRUI Industrial Ltd; St. Hong Kong, China), with detection sensitivity limits of 0.33 μmol/L.

### High performance liquid chromatography (HPLC) for 5-HT, DA and NE level determination

After the rats was euthanized by carbon dioxide inhalation followed by rapid decapitation, the brains were removed and stored at − 80 °C. At the time of detection, 20 μL of 0.1 M HClO_4_ was added to each mg of brain tissue, and then mixed with a cell breaker (Qsonica-Sonicators; St. Melville, NY). The lysates were centrifuged at 20,000 rpm for 20 min 500 μL supernatant was removed, and 125 μL of 0.5 M Na_2_CO_3_, was added and mixed well. The samples were centrifuged for 10 min at 20,000 rpm and then passed through a 0.22-μm filter before sample analysis. All centrifugations were performed at 4 °C.

One day before intervention, the blood of inner canthus was taken, and the blood of femoral artery was collected after intervention. Each time, blood was placed at room temperature for 2 h, before centrifugal at 12,000 rpm 10 min. The supernatants were separated and stored at − 80 °C. Samples of haemorrhagic clearance were taken and melted at room temperature, then 100 mL serum samples were placed in 1.5-mL tubes and isomers were added. A 5% (v/v) perchloric acid solution was added at room temperature for about 10 min to fully precipitate serum protein. Supernatant were centrifuged for 10 min at 12,000 rpm at 4 °C. Supernatant were then transferred into fresh 1.5-mL tubes. Supernatant were centrifuged for 10 min at 12,000 rpm at 4 °C and then filtered through a 0.22-μm membrane for analysis.

The chromatographic conditions were as follows: YMC-pack-ODS-A (100x × 4.0 mm, 3 µm) (YMC CO. LTD; St. Kyoto, Japan) mobile phase: 0.l M potassium dihydrogen phosphate/methanol (85:15, V/V) solution; flow rate: 0.75 mL/min; fluorescence detection excitation and emission wavelengths of 278 and 338 nm, respectively; injection volume: 20 μL; room temperature.

### IL-6, β-EP and BDNF measurement

Blood from the inner canthus were taken one day before CUMS and FA treatment. Femoral artery blood was collected after intervention. Brains were, weighed, frozen in liquid nitrogen, and kept at − 80 °C until assays were performed. The levels of IL-6, β-EP and BDNF in serum and brain tissues were determined by an enzyme linked immunoadsorption determination (ELISA) kit (Wuhan Huamei Biological Technology; St. Wuhan, China) according to the manufacturer’s protocol.

### Statistical analysis

All statistical procedures were performed using SPSS (version 19.0, IBM; Armonk, NY). Data were expressed as mean ± SD, and analysed by one-way analysis of variance (ANOVA) with a Duncan’s test for multiple comparisons. P-values < 0.05 were considered statistically significant.

## Results

### FA increased serum folate and reduced serum homocysteine

**Fig. 2:**
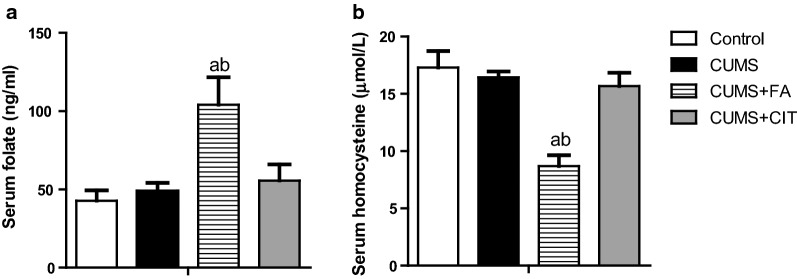
Serum folate (**a**) and homocysteine (**b**) in CUMS rats after 6-week treatment of folic acid, Citalopram or saline. The results are presented as the mean ± SD (n = 8). ^a^*P *< 0.05 compared with Control group; ^b^*P *< 0.05 compared with CUMS group

After 6 weeks of FA treatment in CUMS rats, a significant increase in serum folate and a reduction in serum HCY were observed in CUMS + FA group, compared to CUMS group (P < 0.05, Fig. 2a, b, Additional file 1). Neither serum folate nor HCY were influenced by citalopram.

### Effects of FA on body weight increment

**Fig. 3:**
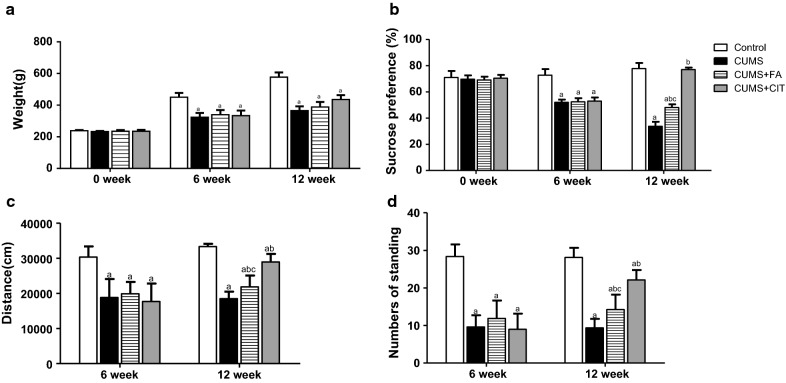
Body weight and depressive-like behaviors of CUMS rats in different group during the experiment. Temporal evolution of body weights on 0, 6th and 12th weeks (**a**). The SPT was conducted and sucrose preference percentage was calculated on 0, 6th and 12th weeks (**b**). OFT was conducted on 6th and 12th weeks. Walking distance (**c**) and standing numbers (**d**) are shown. The results are presented as the mean ± SD (n = 8). ^a^*P *< 0.05 compared with Control group; ^b^*P *< 0.05 compared with CUMS group; ^c^*P *< 0.05 compared with CUMS + CIT group

CUMS rats had a significantly reduced body weight increase compared to the control group (*P* < 0.05). There was no significant difference in body weight increment between CUMS, CUMS + FA and CUMS + CIT groups after 6 weeks of intervention (*P *> 0.05) (Fig. 3a, Additional file 2).

### FA improved the sucrose preference level of CUMS rats

Compared to the control group, the sucrose preference percentage of all rats with CUMS stimulation was significantly reduced on week 6. Compared to CUMS group, FA improved the sucrose preference percentage of CUMS rats significantly (*P *< 0.05). However, this improvement with FA was less pronounced than that with citalopram (*P *< 0.05, Fig. 3b, Additional file 2).

### Effects of FA on the open field test

Stress despair in CUMS rats was assessed by exercise distance and standing times. Exercise distance and the time spent standing were significantly lower in CUMS group than in the control group. After intervention, exercise distance and standing times were significantly higher in the FA group than in CUMS group (*P *< 0.05). Similarly, citalopram exhibited better effects on depressive behaviour than FA (*P *< 0.05, Fig. 3c, d, Additional file 2).

### FA increased DA and NE level but not 5-HT

**Fig. 4:**
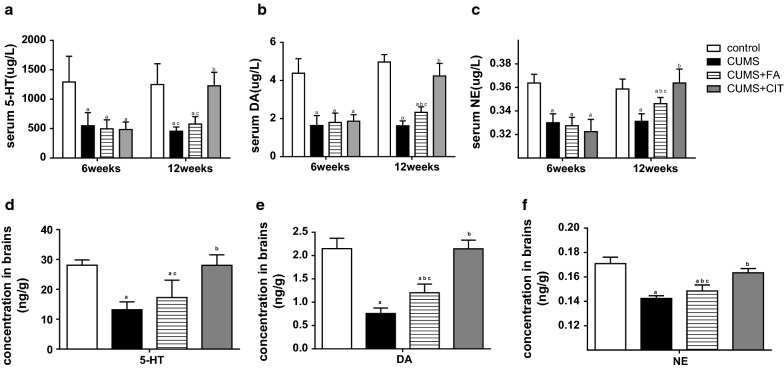
The levels of monoamine neurotransmitter in serum and brains of CUMS rats in different group during the experiment. Serum 5-HT (**a**), DA (**b**) and NE (**c**) were detected on 6th and 12th weeks. The concentration of those three neurotransmitters in brains was tested at the end of the experiment (**d**, **e**, **f**). The results are presented as the mean ± SD (n = 8). ^a^*P *< 0.05 compared with Control group; ^b^*P *< 0.05 compared with CUMS group; ^c^*P *< 0.05 compared with CUMS + CIT group

HPLC was used to detect 5-HT, DA and NE in serum and brains of experimental animals. During CUMS procedure, serum and brain 5-HT, DA and NE levels were significantly decreased (*P *< 0.05, Fig. 4a–e) in CUMS group compared to the control group.

After intervention, DA and NE levels in serum and brain tissue in CUMS + FA group were higher than those in CUMS group (*P* < 0.05, Fig. 4b–f). There was no significant difference in 5-HT levels between CUMS + FA group and CUMS group (*P* > 0.05, Fig. 3a, d). The concentration of all three neurotransmitters in CUMS + CIT group were higher than those in CUMS group (*P* < 0.05, Fig. 4a–f, Additional file 3).

### Effects of FA on IL-6, BDNF and β-EP levels in serum and brains

After 6 weeks of stimulation, IL-6 levels in serum and brain tissue were increased and the levels of BDNF and β-EP were decreased in all groups with CUMS compared to the control group (*P* < 0.05).

**Fig. 5:**
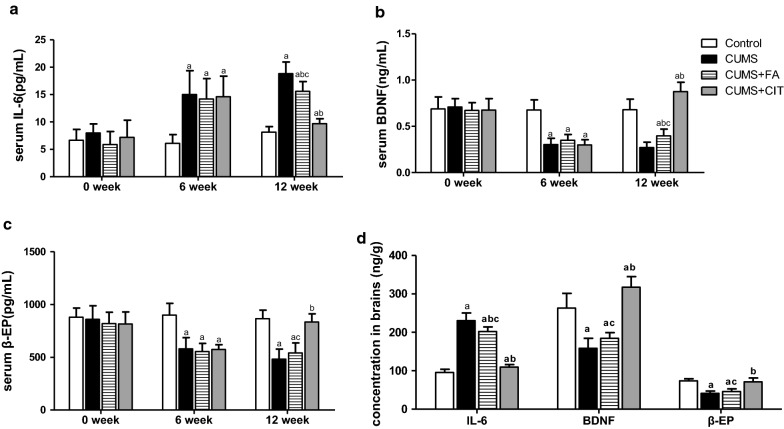
The levels of IL-6, BDNF and β-EP in serum and brains of CUMS rats in different group during the experiment. Serum IL-6 (**a**), BDNF (**b**) and β-EP (**c**) were detected on 0, 6th and 12th weeks. The concentration of those three indicators in brains was tested at the end of the experiment (**d**). The results are presented as the mean ± SD (n = 8). ^a^*P *< 0.05 compared with Control group; ^b^*P *< 0.05 compared with CUMS group; ^c^*P *< 0.05 compared with CUMS + CIT group

Six weeks of FA administration significantly decreased IL-6 levels in serum and brain tissue of CUMS + FA group compared with than of CUMS group (*P* < 0.05, Fig. 5a, d). The level of BDNF in serum but not in brains of CUMS + FA group was higher that that in CUMS group (*P *< 0.05, Fig. 5b, d). However, there was no significant difference in the levels of β-EP in serum and brain tissue between CUMS + FA and CUMS groups (*P* > 0.05, Fig. 5c, d, Additional file 4). Serum and brain tissue IL-6, as well as BDNF and β-EP, was significantly lower in CUMS + CIT group than those in CUMS group (*P* < 0.05, Fig. 5a–d, Additional file 4).

## Discussion

Effective methods for treating depression exist, but remission is not achieved in all cases. Therefore, more and more efforts are being made to optimize adjuvant therapy for current therapies for depression, and the role of FA in depression has been studied as such [17]. The goal of current study was to ascertain the antidepressant-like effects of FA in a rat model of CUMS, then we found that 0.8 mg/kg FA could increase sucrose consumption and exercise activity of OFT.

Several studies have revealed the antidepressant effects of FA, but those treatment doses of FA were much higher than that in our study. The most commonly used dose of FA in other antidepressant animal experiments was 50 mg/kg which equivalent to 476 times of tolerable upper intake level for human adults [8, 18–20]. And 5 mg/kg FA has been shown to improve memory and antioxidant capacity in aged rats [21]. However, the potential risk of excess FA intake is still a concern, the dose used in an animal study should reflect the therapeutic dose used in the clinic.

In clinical research, FA at 5 mg/day showed a significant benefit in female patients with depressive episodes taking 20 mg/day of fluoxetine [22]. FA supplementation at 10 mg/day improved cognitive and depressive states [23]. However, FA supplementation at 0.4 mg/day for 2 consecutive years did not reduce depressive symptoms [24]. Therefore, in our present study, we used 0.8 mg/kg/day of FA, which is closer to the clinical therapeutic dose after conversion to rats, and a significant antidepressant effect was still observed.

Extensive studies show that monoaminergic neurotransmission involving 5-HT, NE and DA exerts major influence on brain circuits by regulating mood, reactivity to psychological stress, self-control, motivation, drive, and cognitive performance [25]. Studies also showed that the levels of DA and 5-HT decreased in the hypothalamic tissue of patients with depression [26]. Folate is necessary for proper biosynthesis of the monoamine neurotransmitters 5-HT, NE and DA [13]. The current results showed that after FA intervention, the levels of NE and DA in brain tissue and serum of CUNS rats increased. The 5-HT levels in this study demonstrated an increasing trend in CUMS + FA group but there was no significant. Therefore, it is speculated that FA can relieve depressive symptoms in CUMS rats by increasing the concentration of certain monoamine neurotransmitters in vivo.

The increase of monoamine neurotransmitters in CUMS rats treated with FA can be explained by a variety of reasons. HCY is toxic to the dopaminergic system in rodents [27]. In addition, folate deficiency and high levels of HCY are associated with decreased levels of 5-hydroxyindoleacetic acid (5-HIAA), a 5-HT metabolite in cerebrospinal fluid which acts as a marker in patients with depression [28, 29]. In the present study, lower serum HCY caused by FA intervention may be one factor in the change in monoamine neurotransmitters in CUMS rats.

Other indicators interacting with monoamine neurotransmitters and playing subtle roles in the pathophysiology of depression were influenced by FA treatment. In the present study, serum and brain IL-6 increased in CUMS rats. Replicated findings confirmed that depression is associated with increased IL-6 [30, 31]. Raised pre-treatment plasma levels of IL-6 was associated with treatment resistance to depression [32, 33]. Treatment with IL-6 has been shown to reduce tetrahydrobiopterin (BH4) content in sympathetic neurons [34], which is a required co-factor of the rate-limiting enzyme for DA synthesis. Therefore, alone with anti-inflammatory effects, FA may also promote DA synthesis by reducing IL-6 content in a depressive rat model.

Clinical and pharmacological studies showed that BDNF concentrations in serum decline in depressive patients, and that antidepressant treatment could induce BDNF expression [35]. The protective effect of BDNF on depression is also associated with monoamine neurotransmitters. For example, BDNF participates in mesencephalic dopaminergic neuron survival [36] and the regulation of the DA system [37]. Furthermore, BDNF can stimulate the growth of 5-HT neurons [38]. NE can induce BDNF expression and activates the phosphatidylinositol 3-kinase (PI3K) and mitogen-activated protein kinase (MAPK) cascades in embryonic hippocampal neurons [14]. In our present study, the increase of serum and brain BDNF and monoamine neurotransmitters after FA treatment further confirmed the interplay between those factors.

As a major endo-opioid substance, β-EP participates in emotional regulation activities such as euphoria and reward behaviour. The increase in serum β-EP in CUMS rats was accompanied by improvement in depressive behavior after electroacupuncture treatment [39]. Injecting β-EP into the ventricles of mice can increase the content of 5-HT in vivo [40]. Although there was a significant difference in serum and brain β-EP in CUMS rats after FA administration, the effect was not as obvious as with citalopram. More evidence is needed in order to reveal the relationship between FA and β-EP.

## Conclusions

These findings indicate that treatment with 0.8 mg/kg FA had an antidepressant-like behaviour in CUMS rats. FA administration led to higher levels of DA and NE in serum and brain tissue, prevented IL-6 release induced by CUMS, and affected the modulation of HCY, BDNF and β-EP. Further studies are required to investigate the effect of FA in depressive patients in the clinic and to determine the central action behind the antidepressant-like effect of FA.

## Supplementary information

**Additional file 1.** Data for Fig. 2 data. Serum folate (A) and homocysteine (B) in CUMS rats after 6-week treatment of folic acid, Citalopram or saline.

**Additional file 2.** Data for Fig. 3. Body weight and depressive-like behaviors of CUMS rats in different group during the experiment.

**Additional file 3.** Data for Fig. 4. The levels of monoamine neurotransmitter in serum and brains of CUMS rats in different group during the experiment. Serum 5-HT (A), DA (B) and NE (C) were detected on 6th and 12th weeks.

**Additional file 4.** Data for Fig. 5. The levels of IL-6, BDNF and β-EP in serum and brains of CUMS rats in different group during the experiment.

## Data Availability

The datasets supporting the conclusions of this article are included within the article and its additional files.

## Abbreviation

CUMS: chronic unpredictable mild stress
HCY: homocysteine
IL-6: interleukin-6
BDNF: brain-derived neurotrophic factor
β-EP: β-endorphin
5-HT: serotonin
NE: norepinephrine
DA: dopamine
SD: Sprague–Dawley
HPLC: high performance liquid chromatography
ELISA: enzyme linked immunoadsorption determination
OFT: the open field test
5-HIAA: 5-hydroxyindoleacetic acid
PI-3K: phosphatidylinositol 3-kinase
MAPK: mitogen-activated protein kinase
